# Quantitative CT Analysis of Pulmonary Ground-Glass Opacity Nodules for the Distinction of Invasive Adenocarcinoma from Pre-Invasive or Minimally Invasive Adenocarcinoma

**DOI:** 10.1371/journal.pone.0104066

**Published:** 2014-08-07

**Authors:** Ji Ye Son, Ho Yun Lee, Kyung Soo Lee, Jae-Hun Kim, Joungho Han, Ji Yun Jeong, O Jung Kwon, Young Mog Shim

**Affiliations:** 1 Department of Radiology and Center for Imaging Science, Samsung Medical Center, Sungkyunkwan University School of Medicine, Seoul, Korea; 2 Department of Pathology, Samsung Medical Center, Sungkyunkwan University School of Medicine, Seoul, Korea; 3 Division of Respiratory and Critical Medicine of the Department of Internal Medicine, Samsung Medical Center, Sungkyunkwan University School of Medicine, Seoul, Korea; 4 Department of Thoracic Surgery, Samsung Medical Center, Sungkyunkwan University School of Medicine, Seoul, Korea; Fundación Jimenez Diaz, Spain

## Abstract

**Objectives:**

We aimed to analyze the CT findings of ground-glass opacity nodules diagnosed pathologically as adenocarcinoma in situ (AIS), minimally invasive adenocarcinoma (MIA), and invasive adenocarcinoma in order to investigate whether quantitative CT parameters enable distinction of invasive adenocarcinoma from pre-invasive or minimally invasive adenocarcinoma.

**Methods:**

We reviewed CT images and pathologic specimens from 191 resected ground-glass opacity nodules with little or no solid component at CT. Nodule size, volume, density, mass, skewness/kurtosis, and CT attenuation values at the 2.5^th^–97.5^th^ percentiles on histogram, and texture parameters (uniformity and entropy) were assessed from CT datasets.

**Results:**

Of 191 tumors, 38 were AISs (20%), 61 were MIAs (32%), and 92 (48%) were invasive adenocarcinomas. Multivariate logistic regression analysis helped identify the 75^th^ percentile CT attenuation value (P = 0.04) and entropy (P<0.01) as independent predictors for invasive adenocarcinoma, with an area under the receiver operating characteristic curve of 0.780.

**Conclusion:**

Quantitative analysis of preoperative CT imaging metrics can help distinguish invasive adenocarcinoma from pre-invasive or minimally invasive adenocarcinoma.

## Introduction

In 2011, the International Association for the Study of Lung Cancer (IASLC), the American Thoracic Society (ATS), and the European Respiratory Society (ERS) proposed a new international multidisciplinary classification system for lung adenocarcinoma [1]. Of special interest to thoracic radiologists and surgeons are the new categories of adenocarcinoma in situ (AIS) and minimally invasive adenocarcinoma (MIA) that represent small (≤3 cm) solitary adenocarcinomas with either pure lepidic growth (AIS) or predominant lepidic growth with ≤5 mm myofibroblastic invasion (MIA) histopathologically. AIS and MIA have been introduced because they should have 100% or near-100% 5-year disease-free survival (DFS), respectively, if completely resected [2]–[5].

Regarding the histopathology of GGNs, the morphologic and textile changes should be thoroughly defined. When GGNs are small and represent atypical adenomatous hyperplasia (AAH) or AIS, tumors grow along the alveolar walls only to appear as homogeneous GGNs at CT [6]. However, with an increase in invasive components (myofibroblastic, not vascular or lymphatic) in MIAs and in invasive adenocarcinomas, the tumors may still appear as GGNs at CT, but may contain portions of regional voxel heterogeneity within the tumor. Thus, MIAs are still seen as a GGN harboring a small central solid component measuring 5 mm or less [7] or pure GGNs of >10 mm in diameter [8]. Even invasive adenocarcinomas may be seen as a large pure GGN greater than 16 mm in diameter [8]. In the case where all three diseases may be seen as GGNs, we hypothesized that an improved CT image-data processing technique would allow us to detect physical voxel-level changes (quantitative CT parameters including uniformity and entropy) within GGNs that could be used to discriminate invasive adenocarcinomas from pre-invasive or minimally invasive lesions [8]. Thus, the aim of the present study was to analyze the CT findings of GGNs diagnosed histopathologically as AIS, MIA, and invasive adenocarcinoma for investigating whether quantitative CT parameter evaluation enables prognostic stratification of the invasive adenocarcinomas from pre-invasive or minimally invasive lesions.

## Methods

Our institutional (Samsung Medical Center [SMC]) review board approved our study (SMC 2011-09-083) with a waiver of informed consent.

### Patients

We (J.Y.S. and H.Y.L.) reviewed the lung cancer surgical registry database of the department of thoracic surgery at Samsung Medical Center (Seoul, Korea) between July 2003 and July 2011 to select patients with persistent GGNs that had been resected completely. We identified 264 patients who underwent complete resection for GGN on CT scans, and 54 patients showing ≥5 mm in diameter of solid component on mediastinal window CT image were excluded after review by two radiologists (J.Y.S. and H.Y.L., with 2 and 11 years of experience, respectively, in thoracic CT interpretation). We excluded 32 patients for radiology- or pathology-related factors: (1) limited quantitative evaluation due to CT images with more than 1.5-mm slice thickness (n = 11) or CT images reconstructed with a bone algorithm (n = 13), (2) insufficient pathologic slides (n = 7), and (3) mucinous type of adenocarcinoma on pathologic review (n = 1). Finally, 178 patients with 191 GGNs with little or no solid component were included in our study.

### Imaging and Analysis

Helical CT images were obtained with 1.25 mm section thickness for transverse images.

CT scans were assessed for tumor size in lung setting/mediastinal setting, density, volume, mass, skewness/kurtosis, and the CT attenuation values at the 2.5^th^, 25^th^, 50^th^, 75^th^, and 97.5^th^ percentiles on the histogram, and texture parameters (uniformity and entropy), independently by two chest radiologist (J.Y.S. and H.Y.L., with 2 and 11 years of experience, respectively, in thoracic CT interpretation), who were unaware of clinical and pathologic results.

For nodule segmentation, tumors were segmented by drawing a region of interest (ROI) covering as large an area as possible from the whole tumor. An ROI was drawn freehand around the tumor using an electronic cursor and mouse. Large vessels and pulmonary arteries were excluded from the ROIs. This process was repeated for each contiguous transverse level, until the entire tumor was covered. Next, voxel-based non-contrast CT numbers were collected from the lesion segmentation.

Heterogeneity within this ROI was quantified by calculating entropy (irregularity) and uniformity (distribution of gray level) [9]. Entropy is a measure of texture irregularity, while uniformity reflects how close the image is to a uniform distribution of the grey levels: higher entropy and lower uniformity represent increased heterogeneity [9]. See Appendix S1 for further details for imaging and analysis.

### Pathologic Evaluation

As for tumor sampling, tumor tissue approximately 10 mm from the entire tumor specimen was placed on a slide. All slides were scanned to produce a high- resolution digital image (0.25 lm/pixel at 40·) using the Aperio Slide Scanning System (ScanScope T3; Aperio Technologies Inc., Vista, CA, USA) [10]. Two experienced lung pathologists jointly interpreted all tissue sections by virtual slides using ImageScope viewing software (Aperio Technologies, Inc.) and a high-resolution monitor [10]. For each case, comprehensive histologic subtyping was performed for the primary tumor in a semi-quantitative manner, to the nearest 5% level, adding up to a total of 100% subtype components per tumor. The extent of invasive component was measured and the most predominant subtype was recorded. When evaluating the predominant pattern, the central fibrosis area and its extent were disregarded. See Appendix S1 for further details for pathologic evaluation.

### Statistical Analysis

For measuring CT variables, the means of values measured by two observers were recorded, and interobserver variability was calculated by using repeated measure data analysis for the intraclass correlation coefficient (ICC). For analysis of all CT variables, the average values of measurement by two reviewers were used. DFS was defined as the time from surgery to recurrence, lung cancer–related death, or last follow-up evaluation. DFS was estimated using the Kaplan–Meier method, with patients followed from time of surgery until recurrence or death from lung carcinoma. Patient demographics and CT parameters were compared among three different pathologic subtypes (e.g., AIS, MIA and invasive adenocarcinoma) by using one-way ANOVA with post hoc test of Bonferroni. Bonferroni correction was also used to account for multiple comparisons. As for multiple GGNs in a patient, we did not take into account within-patient correlation because each of them was considered as an independent synchronous lesion [11]. A multivariate logistic regression analysis was used to identify the independent factors to predict invasive adenocarcinoma from minimally or pre-invasive adenocarcinoma (AIS), for which characteristics with a *P* value of less than 0.10 using one-way ANOVA were all used as the input variables for logistic regression analysis. In logistic regression analysis, a backward stepwise selection mode was used, with iterative entry of variables on the basis of test results (*P*<0.05). The removal of variables was based on likelihood ratio statistics with a probability of 0.10. Also, for multivariate analysis, logistic regression analysis was used with multi-colinearity examination by using the variance inflation factor (VIF). Spearman correlation analyses were performed to evaluate the correlation between the extent of the invasive component and all imaging variables. Finally, ROC analysis was performed to evaluate the differentiating performance of logistic regression models in discriminating invasive adenocarcinoma from AIS or MIA. Statistical significance was evaluated with software (SPSS, version 19.0, 2010; SPSS, Chicago, Ill). A P value less than 0.05 was considered to indicate a statistically significant difference.

## Results

### Patient Population and Surgical Outcomes

Of 191 tumors, 38 were AIS (20%), 61 were MIA (32%), and 92 were invasive adenocarcinoma (48%). Subtype classification of the 92 invasive adenocarcinomas resulted in 49 lepidic-predominant (53%), 40 acinar-predominant (43%), and three papillary-predominant (3%) adenocarcinomas. None had lymphatic, vascular, perineural or pleural invasion. The median extent of invasion in 92 invasive adenocarcinomas was 9.8 mm (range, 5.1–19.7 mm). CT findings were pure GGNs without solid component in 156 tumors. Of 35 tumors showing <5 mm solid component on CT images, three were AISs (9%), eight were MIAs (23%), and 24 were invasive adenocarcinomas (69%). The solid component on CT images of GGNs in three AISs corresponded histopathologically to the area of collapse or severe narrowing of alveolar air spaces. In five of eight MIAs, the solid component on CT images was caused histologically by prominent central scar tissue, and in the remaining three MIAs, it was due to alveolar airspace collapse. In 24 invasive adenocarcinomas, the solid component was caused by myofibroblastic invasive component (n = 21), prominent central scar tissue (n = 2), or centrally located bronchovascular structures (n = 1).

**Figure 1:**
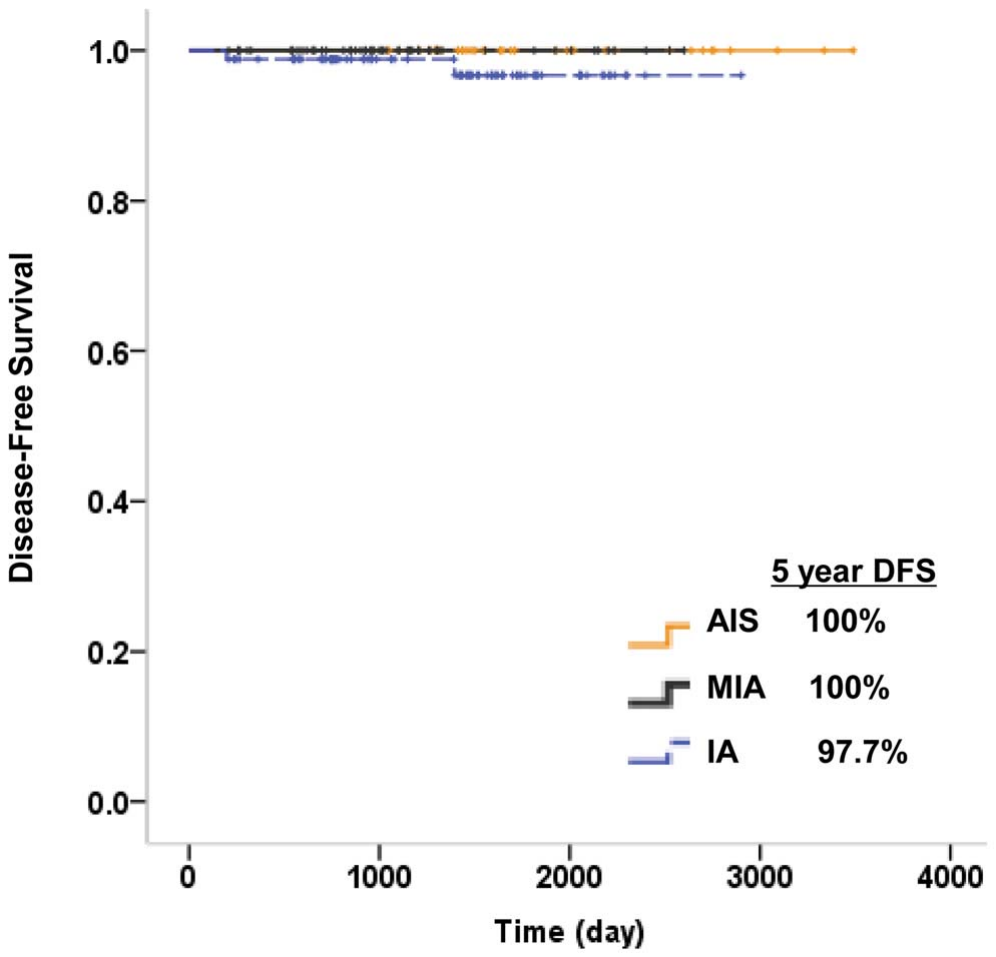
DFS curves for AIS, MIA and invasive adenocarcinoma (IA) groups.

All 191 tumors were removed via either sublobar resection (wide wedge resection or segmentectomy) (81 nodules) or lobectomy (110 nodules). The median follow-up period after surgical resection for all patients was 44 months (range, 7–116 months). Forty-one patients (23%) had a follow-up period of more than five years, and 10 patients (5.6%) had a follow-up period of less than one year. By January 2013, two patients who underwent lobectomy for invasive adenocarcinomas developed recurrent disease after surgical resection. One had pulmonary metastasis and the other had metastases to bone. Five-year DFS was 100% for both AISs and MIAs, while it was 97.7% for invasive adenocarcinomas (Fig. 1).

### Comparison between three different lung adenocarcinomas based on the degree of invasive component

Interobserver agreement for measurements of the size was high, whereas agreement for the tumor volume and mass was moderate. Intraclass correlation coefficients were 0.96 (95% confidence interval [CI]: 0.94–0.98) for size in the lung setting, 0.99 (95% CI: 0.99–1.00) for size in the mediastinal setting, 0.75 (95% CI: 0.68–0.81) for tumor volume, and 0.69 (95% CI: 0.63–0.75) for tumor mass. Table 1 shows comparisons of all CT parameters according to three pathologic subtypes. Patient sex showed no differences among the three subtypes on univariate analysis.

**Table 1:** Characteristics of lung adenocarcinoma with little solid component on CT in reference to invasion status (n = 191).

Variable	Adenocarcinoma In Situ (AIS) (n = 38)	Minimally Invasive Adenocarcinoma (MIA) (n = 61)	Invasive Adenocarcinoma (n = 92)	*P*	*P1*	*P2*	*P3*
Age (y)	59.3±10.0	57.7±9.3	62.8±9.7	**0.01**	0.95	**0.01**	0.23
Male-to-female ratio**	21: 17	21: 28	49: 55	0.21			
CT parameters							
Size (mm)							
In lung setting	13.2±6.7	15.2±6.1	18.3±6.0	**<0.01** *	0.84	0.26	**<0.01** *
In mediastinal setting	0.37±1.26	0.61±1.57	1.26±2.25	**0.04** *	>0.99	0.24	0.08
Volume (cm^3^)	0.25±0.29	0.41±0.43	0.50±0.54	**0.04** *	0.42	0.74	**0.03** *
Density	0.33±0.13	0.39±0.12	0.43±0.12	**<0.01** *	0.10	0.12	**<0.01** *
Mass (g)	0.10±0.13	0.16±0.16	0.21±0.27	**0.02** *	0.59	0.28	**0.02** *
Histogram analysis							
Skewness	0.42±0.50	0.48±0.50	0.43±0.51	>0.99			
Kurtosis	3.12±1.61	2.85±1.10	2.76±0.96	>0.99			
2.5^th^ percentile (HU)	−878±58.4	−861±59.4	−865±54.0	>0.99			
25^th^ percentile (HU)	−761±82.6	−742±76.9	−725±93.9	0.98			
50^th^ percentile (HU)	−667±112	−632±101	−596±118	**0.03** *	>0.99	>0.99	**0.03** *
75^th^ percentile (HU)	−562±152	−494±142	−445±141	**<0.01** *	0.49	0.70	**<0.01** *
97.5^th^ percentile (HU)	−370±209	−248±198	−173±177	**<0.01** *	**0.049** *	0.35	**<0.01** *
Texture analysis							
Uniformity	0.0077±0.0045	0.0059±0.0022	0.0044±0.0020	**<0.01** *	**0.01** *	**<0.01** *	**<0.01** *
Entropy	7.42±0.86	7.68±0.65	8.14±0.64	**<0.01** *	0.18	**<0.01** *	**<0.01** *

Note—Classified According to the International Multidisciplinary Lung Adenocarcinoma Classification system.

Unless otherwise indicated, data are means ± standard deviation.

**Data number of individuals.

*P<0.05

* *P* values were calculated with one-way ANOVA.

In terms of size variables and histogram analysis variables, *P* values are Bonferroni-corrected *P* values (Bonferroni-correction, *P*<0.05÷2 for size variables and *P*<0.05÷7 for histogram variables).

*P*1 indicates the *P* values for post hoc analyses of AIS versus MIA.

*P*2 indicates the *P* values for post hoc analyses of MIA versus invasive adenocarcinoma.

*P*3 indicates the *P* values for post hoc analyses of AIS versus invasive adenocarcinoma.

**Figure 2:**
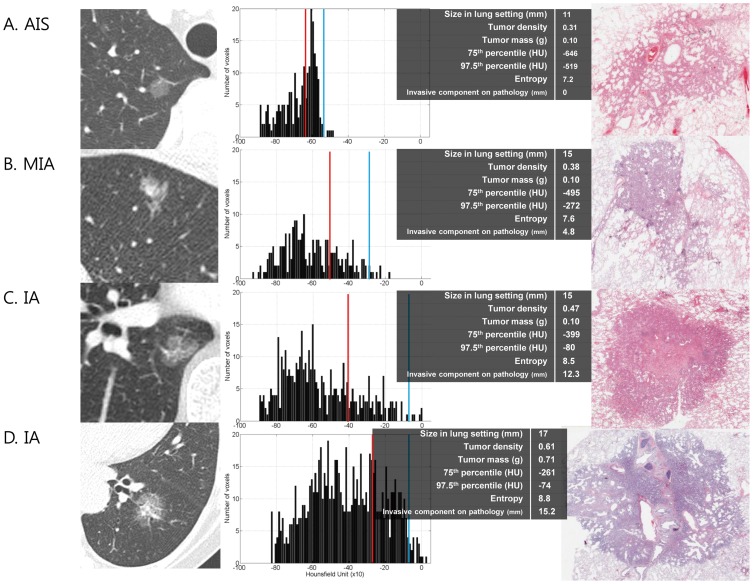
CT image, histogram distribution of CT attenuation value, and photomicrograph (hematoxylin-eosin stain; original magnification, X 40). (A) is a case of AIS, (B) is a case of MIA, (C) and (D) are cases of invasive adenocarcinoma. First three cases show pure GGNs without a solid component, whereas (D) shows GGN with 2 mm-solid component on CT image. As for histogram distribution, the vertical axis in each histogram shows the number of pixels in the segmented tumor. The red and blue lines indicate the values for 75^th^ and 97.5^th^ percentile. The horizontal axis shows the CT attenuation values. As compared with histograms of (A) AIS and (B) MIA, those of (B) MIA and (C) invasive adenocarcinoma show increased values in the 75^th^ and 97.5^th^ percentile. Tumor density was also increased, whereas tumor mass showed no difference. Histogram of MIA demonstrates a flat peak with high entropy as compared with that of AIS. Histogram graph of (D) shows two peaks, which is different from one peak of (A),(B), and (C). In a photomicrograph of (A) AIS, this circumscribed nonmucinous tumor grows purely with a lepidic pattern. No foci of invasion or scarring are seen. A photomicrograph of (B) MIA consists primarily of lepidic growth with a small (4.8 mm) upper area of acinar invasion. A photomicrograph of (C) invasive acinar adenocarcinoma consists of round to oval-shaped malignant glands invading a fibrous stroma 7 mm in length and a smaller area of lepidic growth only at the tumor periphery. Another photomicrograph of invasive acinar adenocarcinoma (D) shows centrally located bronchus, which is the main cause of solid component of CT image.

On multivariate analysis, nodule volume showed multi-colinearity (VIF >10) on variance inflation factor analysis, and thus, volume was removed from the multivariate analysis. Logistic regression analysis (Table 2 and Fig. 2) demonstrated that the 75th percentile CT attenuation value and entropy were independent predictors of invasive adenocarcinomas (odds ratio [OR] = 1.04, P = 0.04, and OR = 3.40, P<0.01, respectively).

**Table 2:** Multivariate analysis for stratification among AIS, MIA and invasive adenocarcinoma.

	Invasive adenocarcinoma vs. others (AIS or MIA)		
Variable	Odds Ratio	95% CI	*P*
Age (y)	1.02	0.99–1.06	0.21
Size in lung setting (mm)	1.03	0.94–1.14	0.50
Size in mediastinal setting (mm)	1.01	0.80–1.28	0.91
Density	0.82	0.09–7.87	0.33
Mass (g)	0.03	0.00–343.21	0.45
50^th^ percentile (HU)	1.02	0.99–1.06	0.27
75^th^ percentile (HU)	1.04	1.01–1.96	**0.04** *
97.5^th^ percentile (HU)	1.01	0.99–1.02	0.10
Uniformity	0.03	0.00–342.21	0.45
Entropy	3.40	2.05–5.64	**<0.01** *

Note.— *CI* confidence interval.

* *P*<0.05.

### Correlation between imaging parameters and pathology

Relationships between all parameters and extent of invasion on pathology are shown in Table 3. Tumor size in mediastinal setting, volume, density, mass, the CT attenuation values at the 25^th^, 50^th^, 75^th^, and 97.5^th^ percentiles on the histogram, and entropy correlated positively with extent of invasion with statistical significance, whereas uniformity was negatively correlated.

**Table 3:** Correlation of imaging biomarker features with extent of invasion on pathology.

Imaging variable	*R^+^*	*P*
Size In mediastinal setting (cm)	**0.33** **	**<0.01**
Volume (cm^3^)	**0.20** **	**<0.01**
Density	**0.28** **	**<0.01**
Mass (g)	**0.23** **	**<0.01**
Skewness	−0.01	0.89
Kurtosis	−0.10	0.16
2.5^th^ percentile (HU)	0.004	0.95
25^th^ percentile (HU)	**0.17** *	**0.02**
50^th^ percentile (HU)	**0.23** **	**<0.01**
75^th^ percentile (HU)	**0.29** **	**<0.01**
97.5^th^ percentile (HU)	**0.34** **	**<0.01**
Uniformity	**−0.44** **	**<0.01**
Entropy	**0.49** **	**<0.01**

Note—^+^Data are Spearman correlation coefficients.

* *P*<0.05.

** *P*<0.01.

### Predictive probability of quantitative CT parameters for pathologic classification (invasive adenocarcinoma from AIS or MIA)

**Figure 3:**
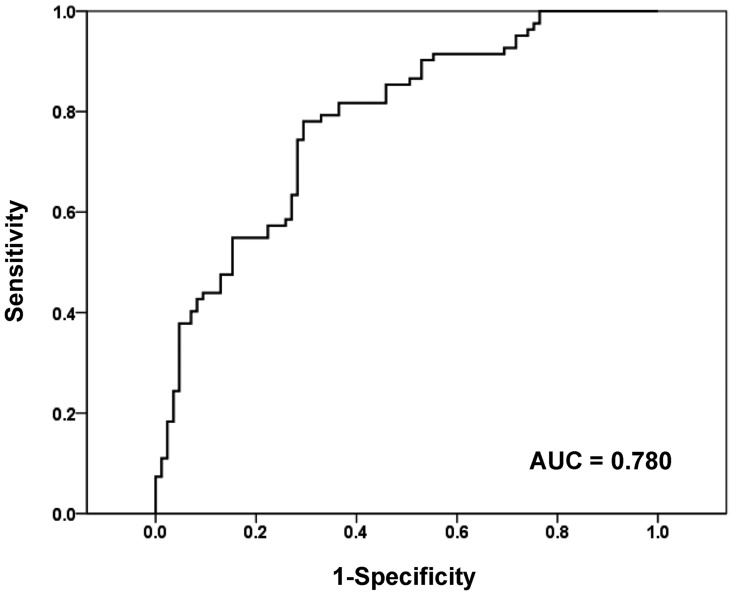
Receiver operating characteristic (ROC) curve for predicting invasive adenocarcinoma with imaging parameters. For invasive adenocarcinoma prediction, ROC curve based on the combination of the 75^th^ percentile CT attenuation value and entropy also shows significant diagnostic accuracy (AUC, 0.780).

Based on multivariate analysis, we investigated whether we could accurately identify invasive adenocarcinoma by reversely combining significant predictive factors (Fig. 3). As for the prediction of invasive adenocarcinoma, when two significant factors were combined (combination of the 75th percentile CT attenuation value on histogram ≥−470 HU and entropy ≥7.90), the AUC value of ROC was 0.780 (95% CI: 0.711–0.849, P<0.01), and 60 of 69 (87%) GGNs, which met both requirements, were correctly predicted to be invasive adenocarcinoma.

## Discussion

We demonstrated that histogram analysis of conventional CT imaging metrics enables the differentiation of invasive adenocarcinoma from AIS or MIA among lesions that appear as GGN with little solid component on CT scans. The histogram approach provides a more comprehensive assessment of the tissue, especially when the distribution is not normal, and it is sensitive in the detection of tumor heterogeneity. It should be noted that most imaging variables used to compare tumor subtypes, such as tumor volume, density, mass, skewness or kurtosis when corrected for multivariate analysis, failed to achieve statistical significance. Thus, our results suggest that the proposed histogram analysis of CT imaging parameters provides higher discriminatory ability than that with information from conventional CT imaging, and aids in improved classification of lung adenocarcinoma of pure GGNs.

There are several issues regarding the management algorithm of pure GGN. First, the classification of AIS, MIA and invasive adenocarcinoma is basically based on pathologic specimens during the surgery or after resection. By contrast, frozen histologic examination for intraoperative pathology diagnosis is sometimes not sufficient to determine three histopathologic categories owing to severe architectural distortion and complete collapse of the alveolar spaces during cryosection [12]. Therefore, for the surgical resection of a tumor to be performed adequately, a precise pre-operative diagnosis may be critical for choosing the resection boundary of the tumor. Second, several studies have reported that pathologic invasive features are not rare, even in small and pure GGNs [6], [13]–[17]. Therefore, histologic prediction of GGNs by qualitative visual assessment alone may miss the pathologic invasive component of pure GGN adenocarcinomas. In other words, some studies revealed pure GGNs on thin-section CT may show invasive adenocarcinoma on histopathology [18], in which stromal or myofibroblastic invasion of 5 mm or smaller in MIA or even of greater than 5 mm in well-differentiated invasive adenocarcinoma may manifest as pure GGN on high-resolution CT (HRCT) because of the limited resolution (200–300 mm) of HRCT images [18].

This study showed that measuring 75^th^ percentile CT attenuation value in GGNs with little solid component can help differentiate invasive adenocarcinomas from pre-invasive adenocarcinomas (AIS or MIA). The reasons why this 75^th^ percentile was superior to the tumor density or tumor mass could be enumerated as follows: (1) three different subgroups of AIS, MIA and invasive adenocarcinoma are all shown as GGNs with little solid component on CT scan, so the mean value for density or mass may frequently be similar among the subgroups; (2) because the 75^th^ percentile indicates the high CT attenuation value zone within the tumor, differences in the thickening of alveolar septa and cellularity among AIS, MIA, and invasive adenocarcinoma may be disclosed. Even in cases where the average value like tumor density or mass lacked sufficient sensitivity to differentiate three different subgroups, the value from the high CT attenuation zone could have local variation with more sensitive preservation of spatial information. This result might be anticipated based on the fact that an increasing CT attenuation value from the 50^th^ to 97.5^th^ percentile could enhance differentiation in Table 1. Similar results were reported previously by other authors [19]. Ikeda reported that the values of the 75^th^ percentile of AAH, AIS, and adenocarcinoma show significant differences between AAH and AIS, and between AIS and adenocarcinoma (*P*<0.05).

We observed significantly reduced uniformity and increased entropy in a step-by-step fashion from AIS to MIA and invasive adenocarcinoma, and entropy remained an independent predictor for invasive adenocarcinoma after multivariate analysis. Several studies have suggested that increased heterogeneity (higher entropy, lower uniformity) is associated with malignancy in non–small cell lung cancer [20], colorectal cancer [21] and renal cell cancer [9]. In a study of 17 patients with non–small cell lung cancer [20], unenhanced CT texture analysis and resultant coarse texture uniformity also correlated negatively with tumor stage. According to the natural chronologic evolution of a lung cancer manifesting as a pure GGN on CT, it is generally accepted that a GGN increases in size, then the solid portion within the lesion tends to appear, and finally the solid portion increases in extent [22]. Heterogeneous changes in entropy and uniformity in our study reflect well this evolution of the malignant progress. To the best of our knowledge, the present study is the first study of the relationship between texture features of CT images in lung adenocarcinoma and histologic subtypes. In addition, at the beginning of the present study, we expected that skewness or kurtosis would help distinguish the three subgroups like other tumors [23], [24]. However, the distinction was not accomplished with skewness and kurtosis. We presume that the patterns of the histogram graphs of AIS, MIA and invasive adenocarcinoma might vary too much to provide separation among the subgroups.

The present study demonstrates that even in patients with invasive adenocarcinoma, for which the median extent of invasion was 9.8 mm (range, 5.1–19.7 mm), 97.7% (90 of 92 patients) had DFS for 5 years. A good prognosis of invasive adenocarcinoma shown as pure GGN may be explained, in part, by the difference in the predominant subtypes. All invasive adenocarcinomas in our study were lepidic, acinar or papillary predominant tumors, which are known to show good prognosis as compared with micropapillary or solid predominant tumors. Travis et al. [1] concluded that all histologic subtypes other than lepidic predominant adenocarcinoma show solid nodules on CT. However, as seen in Fig. 2, well-organized and well-differentiated acinar or papillary predominant adenocarcinomas can also be seen as pure GGNs.

Our study was limited inherently by its retrospective design, and we may have had a selection bias. However, we tried to include as many patients as possible for whom the pathologic assessment of the whole tumor was feasible. We also included GGNs with ≤5-mm solid component on CT scans as well as pure GGNs with the insight that the nonmucinous type of MIA can appear as a part-solid nodule consisting of a predominant ground-glass component and a small central solid component measuring 5 mm or less [1]. As a result, we excluded the patients for whom only a small fragment of a tumor was available for diagnosis only or in whom the entire tumor was not available for surgical reasons, and 3 AIS and 8 MIAs having <5 mm solid component could be included for analysis. Another potential limitation is that the pathologic invasive component was evaluated in a subjective manner. Thunnissen et al. [25] assessed the reproducibility of invasion of lung adenocarcinoma among an international group of pulmonary pathologists, and concluded that there is fair reproducibility distinguishing invasive from in-situ (wholly lepidic) patterns. Nevertheless, we tried to reduce inter-observer and intra-observer variability by using virtual microscopy [10]. Recent related studies showed that virtual microscopy is a reliable and more reproducible technology [10], [26]–[28]. Also in our study, digital pathology offered a rigorous and reproducible method for quantifying invasive and noninvasive components of histopathology.

In conclusion, quantitative analysis of CT imaging metrics can help distinguish invasive adenocarcinoma from pre-invasive or minimally invasive adenocarcinoma shown as GGN with little solid component on CT scans.

## Supporting Information

Appendix S1 (DOC)
