# Supplementary material for: Quantitative CT Analysis of Pulmonary Ground-Glass Opacity Nodules for the Distinction of Invasive Adenocarcinoma from Pre-Invasive or Minimally Invasive Adenocarcinoma
Source: PLoS One. 2014 Aug 7;9(8):e104066. doi: 10.1371/journal.pone.0104066 (PMC4125172; doi:10.1371/journal.pone.0104066)
Supplement: Appendix S1 — (DOC) [file pone.0104066.s001.doc]

**Appendix S1**

**Imaging and Analysis**

CT scans were obtained with various scanners, but mainly with Light Speed16 (16 detector-row) and Light Speed VCT (64 detector-row) scanners (GE Medical Systems, Milwaukee, Wisconsin). Unenhanced CT images were obtained with the following parameters: detector collimation, 0.625 mm; field of view, 34.5 cm; beam pitch, 1.375; gantry speed, 0.6 second per rotation; 120 kVp; 150–200 mA; and section thickness, 1.25 mm for transverse images. All imaging data were reconstructed using soft-tissue algorithms. CT data were sent directly to a picture archiving and communication system (Path-Speed or Centricity 2.0; GE Healthcare, Mt. Prospect, Ill). The monitors were used to view both mediastinal (width, 400 HU; level, 20 HU) and lung (width, 1500 HU; level, -700 HU) window images.

To determine the size of the whole lesion on lung window images (width, 1500 HU; level, -700 HU), we independently identified the slice that showed the maximum diameter of the nodule on axial sections and measured the maximum diameter of the nodule using an electronic cursor manually. For the size of the solid part, the same approach was used on the mediastinal window images (width, 400 HU; level, 20 HU).

For tumor density and volume, the computer automatically calculated the density from mean attenuation of total voxels and volume by multiplying the number of voxels by the unit volume of a voxel [1]. Tumor mass (in grams) was calculated by multiplying tumor volume (in cubic centimeters) by mean tumor density [2, 3].

Texture parameters (entropy and uniformity) are defined below where l is the number of grey levels (e.g., l

=

1 to k, indicating a grey level from 1 to k) and p(l) is the probability of the occurrence of the grey level l based on the image histogram technique:


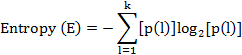


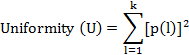


A histogram of the voxel-based CT numbers was displayed, and we computed CT attenuation values at the 2.5^th^, 25^th^, 50^th^, 75^th^, and 97.5^th^ percentiles of pixel attenuation value (Hounsfield Unit, HU) on the histogram of the sorted values (*e.g.*, a 25^th^ percentile value means a CT attenuation value for 25% of pixels calculated from the pixel with the minimum CT attenuation value, and a 50^th^ percentile signifies the median pixel CT attenuation value) [4].

Additionally, a spreadsheet of all of the values was used to compute histogram distribution parameters, kurtosis and skewness offline [5]. Skewness describes the degree of asymmetry of a histogram; a histogram with a long tail to the right has a positive skewness value, and a perfectly symmetric distribution has a skewness value of zero. Kurtosis describes the peak sharpness of a histogram; a histogram that has a sharper peak than a normal distribution has a positive kurtosis value, and a normal distribution has a kurtosis of zero. To measure tumor density and volume, and to analyze texture and histogram from CT data in a tumor region, custom software was implemented using MATLAB R2009a (The Mathworks, Natick, MA).

**Pathologic Evaluation**

Each resected specimen (entire tumor) was evaluated with standard pathologic methods as described in the surgical pathologic dissection manual of the Department of Pathology [6]. All resected specimens were designated R0 (no residual tumor at the primary tumor site after surgical resection).

Two experienced lung pathologists (J.Y.J. and J.H. with 6 and 18 years of experience in lung pathology), jointly interpreted all tissue sections by virtual slides using ImageScope viewing software (Aperio Technologies, Inc.) and a high-resolution monitor [7]. For each case, the specimens were reviewed according to IASLC/ATS/ERS International Multidisciplinary Lung Adenocarcinoma Classification criteria [8].

**References**

1. Kim EA, Johkoh T, Lee KS et al (2001) Quantification of ground-glass opacity on high-resolution CT of small peripheral adenocarcinoma of the lung: pathologic and prognostic implications. AJR Am J Roentgenol 177:1417-1422

2. Mull RT (1984) Mass estimates by computed tomography: physical density from CT numbers. AJR Am J Roentgenol 143:1101-1104

3. Lee HY, Jeong JY, Lee KS et al (2012) Solitary pulmonary nodular lung adenocarcinoma: correlation of histopathologic scoring and patient survival with imaging biomarkers. Radiology 264:884-893

4. Ikeda K, Awai K, Mori T, Kawanaka K, Yamashita Y, Nomori H (2007) Differential diagnosis of ground-glass opacity nodules: CT number analysis by three-dimensional computerized quantification. Chest 132:984-990

5. Chandarana H, Rosenkrantz AB, Mussi TC et al (2012) Histogram analysis of whole-lesion enhancement in differentiating clear cell from papillary subtype of renal cell cancer. Radiology 265:790-798

6. SC. L (2006) Manual of surgical pathology. 2nd ed., Elsevier Churchill Livingstone, New York, NY

7. Ozluk Y, Blanco PL, Mengel M, Solez K, Halloran PF, Sis B (2012) Superiority of virtual microscopy versus light microscopy in transplantation pathology. Clin Transplant 26:336-344

8. Travis WD, Brambilla E, Noguchi M et al (2011) International association for the study of lung cancer/american thoracic society/european respiratory society international multidisciplinary classification of lung adenocarcinoma. J Thorac Oncol 6:244-285
